# Supplementary figures and images for: Octopamine signaling from clock neurons plays dual roles in Drosophila long-term memory
Source: PLoS Genet. 2026 Feb 17;22(2):e1012045. doi: 10.1371/journal.pgen.1012045 (PMC12923120; doi:10.1371/journal.pgen.1012045)

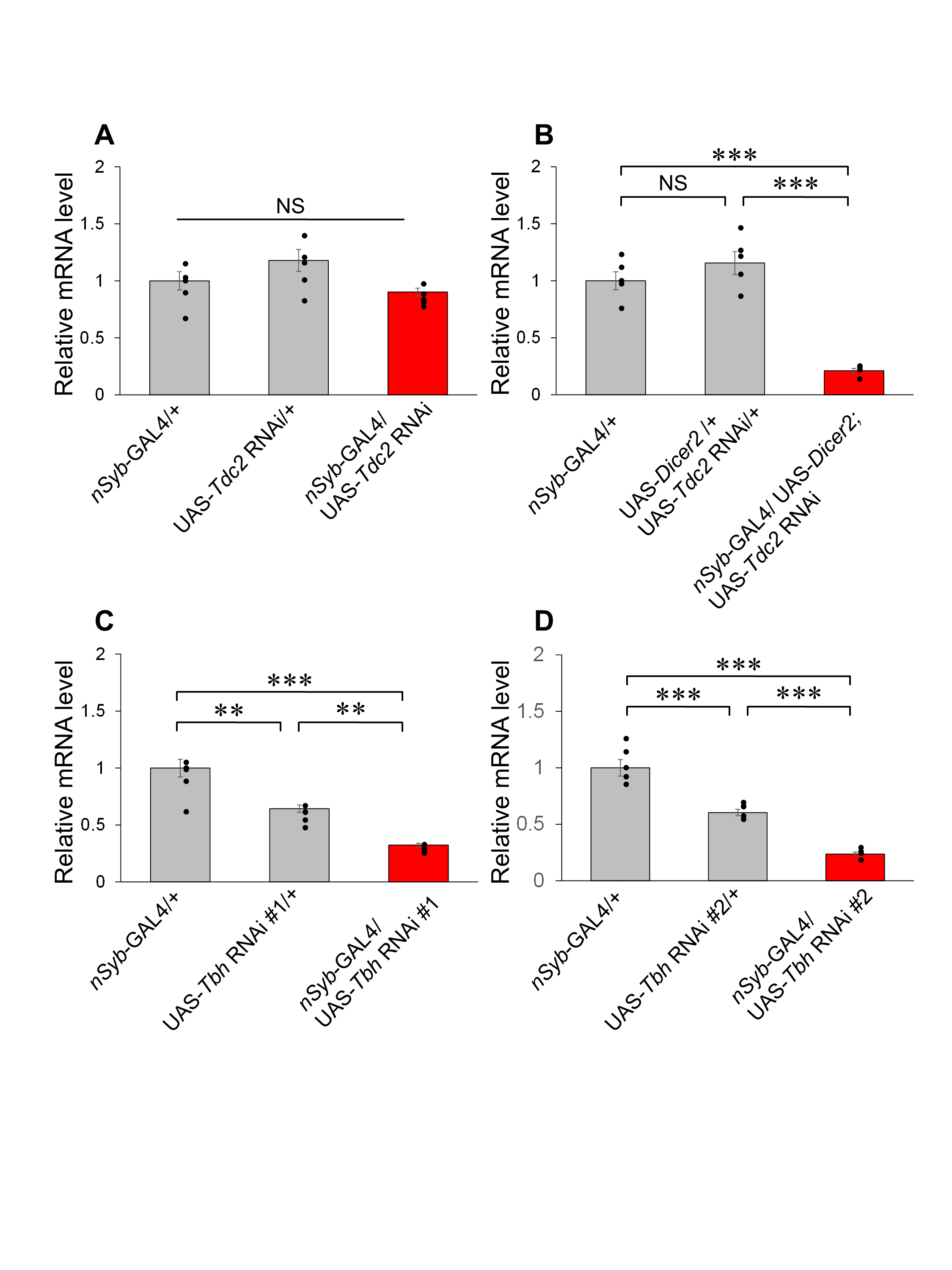

Supplement: S1 Fig — (A–D) A pan-neural GAL4 line, nSyb-GAL4, was used to knockdown Tdc2 or Tbh in the experiments. nSyb-GAL4/ + males were used as the control. Mean ± SEM was calculated from five replicates. We visualized the data using a bar chart with individual data points (black circles). NS, not significant. N = 5 in each bar. **, P < 0.01; ***, P < 0.001; NS, not significant. (A) UAS-Tdc2 RNAi, (B) UAS-Dicer2; UAS-Tdc2 RNAi, (C) UAS-Tbh RNAi #1, and (D) UAS-Tbh RNAi #2 were used in the experiments. (TIF) [file pgen.1012045.s001.TIF]

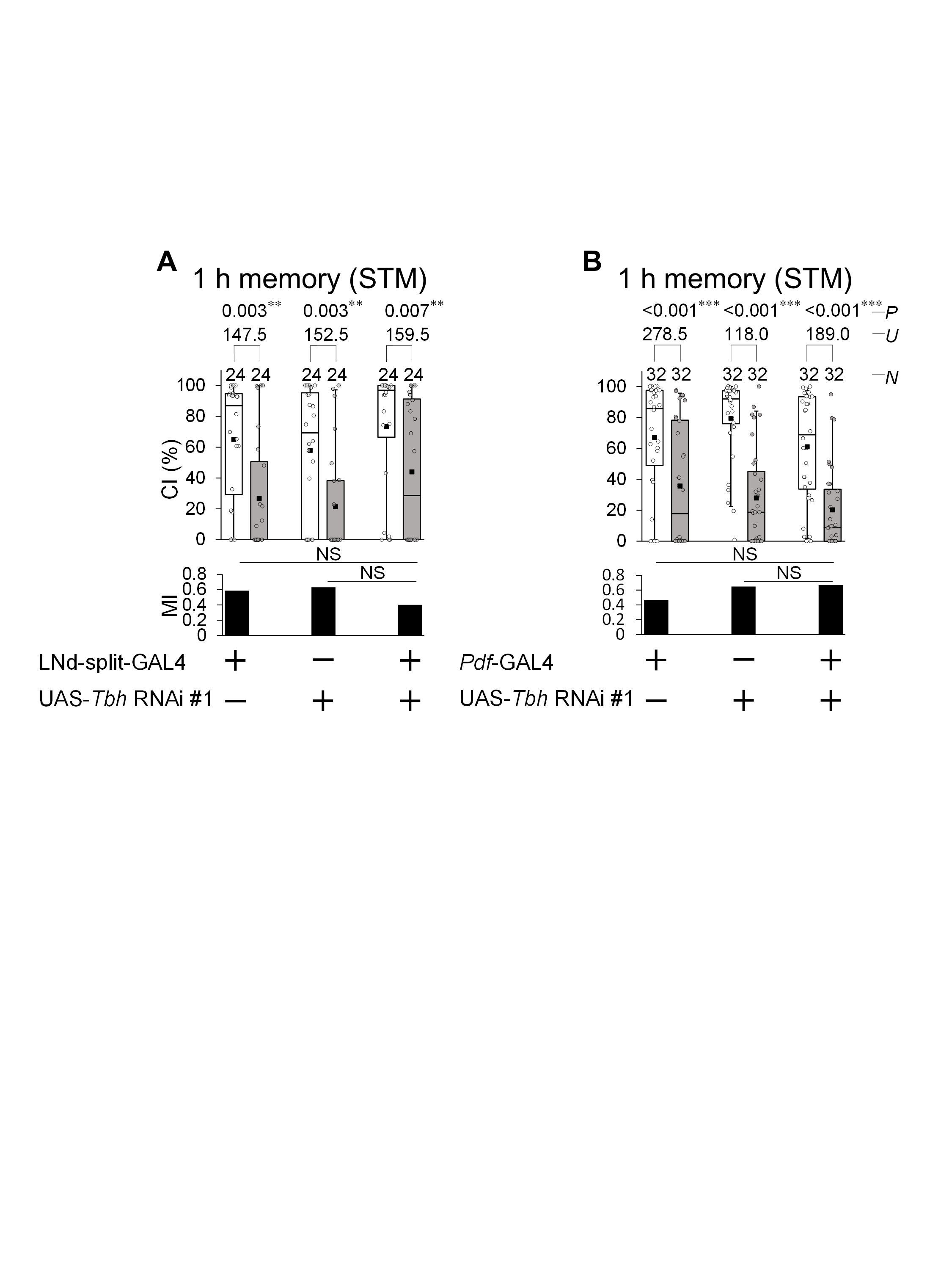

Supplement: S2 Fig — (A) LNd-split-GAL4/UAS-Tbh RNAi #1 males were used in the experiments. LNd-split-GAL4/+ and UAS-Tbh RNAi #1/ + males were used as the control. (B)Pdf-GAL4/UAS-Tbh RNAi #1 males were used in the experiments. Pdf-GAL4/+ and UAS-Tbh RNAi #1/ + males were used as the control. (A, B) Males in each genotype were tested 1 h after 1 h conditioning. We visualized the data using a box plot with individual data points (white circles). See Fig 1 for an explanation of box plots. **, P < 0.01; ***, P < 0.001; P, probability; U, Mann–Whitney U; N, sample size in each box. (TIF) [file pgen.1012045.s002.TIF]

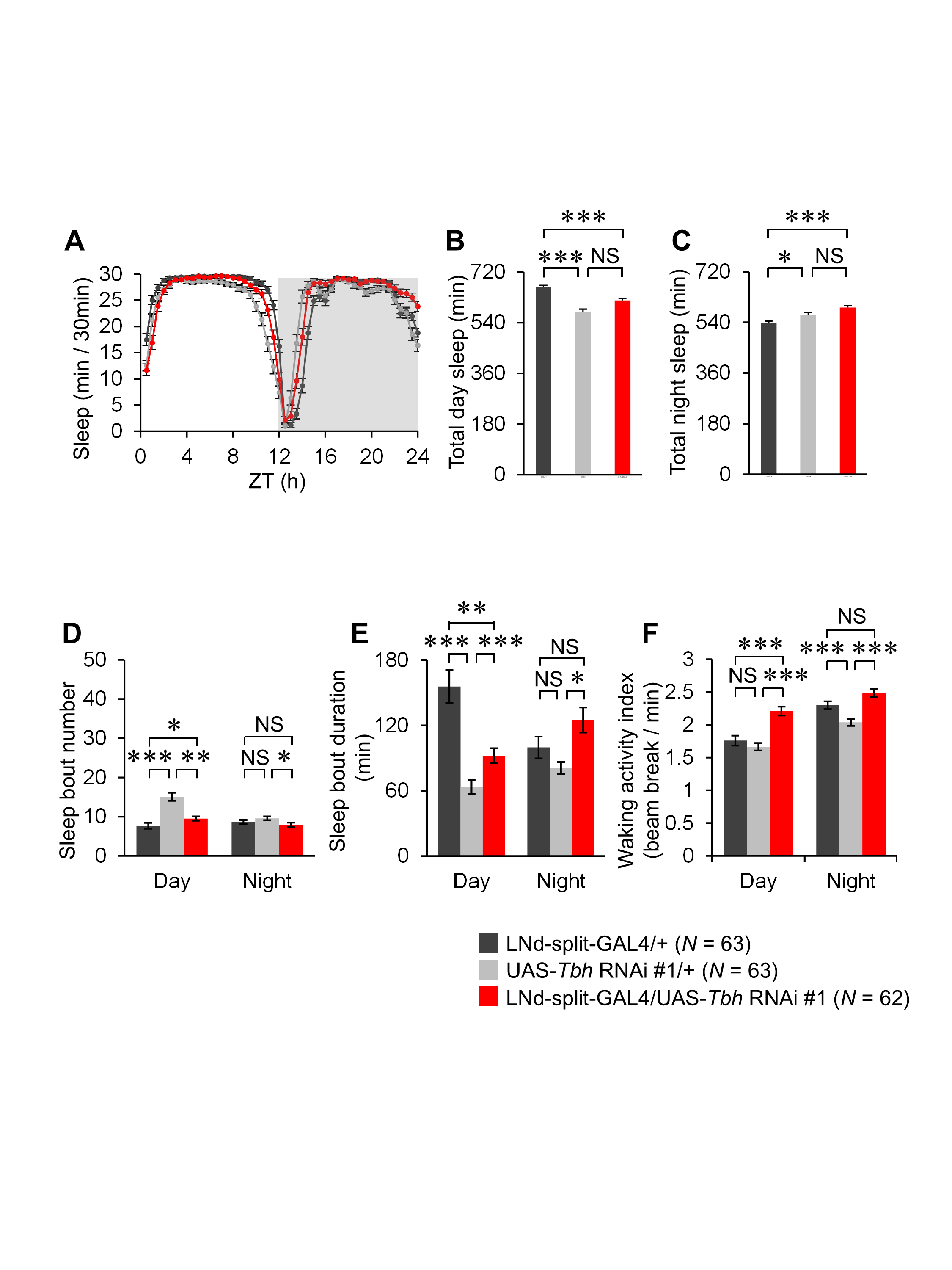

Supplement: S3 Fig — All sleep/wake parameters (daily sleep pattern, total day sleep, total night sleep, sleep bout number, sleep bout duration, waking activity index) were analyzed using the data averaged over 3 days of LD. (A–F) Error bars show SEM in each figure. Black circles and bars, LNd-split-GAL4/ + ; gray circles and bars, UAS-Tbh RNAi #1/ + ; red circles and bars, LNd-split-GAL4/ UAS-Tbh RNAi #1. *, P < 0.05; **, P < 0.01; ***, P < 0.001; NS, not significant. (A) Daily sleep patterns of control and experimental flies. (B) Total sleep amount during the day. (C) Total sleep amount during the night. (D) Sleep bout number during day and night. (E) Sleep bout duration during day and night. (F) Waking activity indices during day and night. (TIF) [file pgen.1012045.s003.TIF]

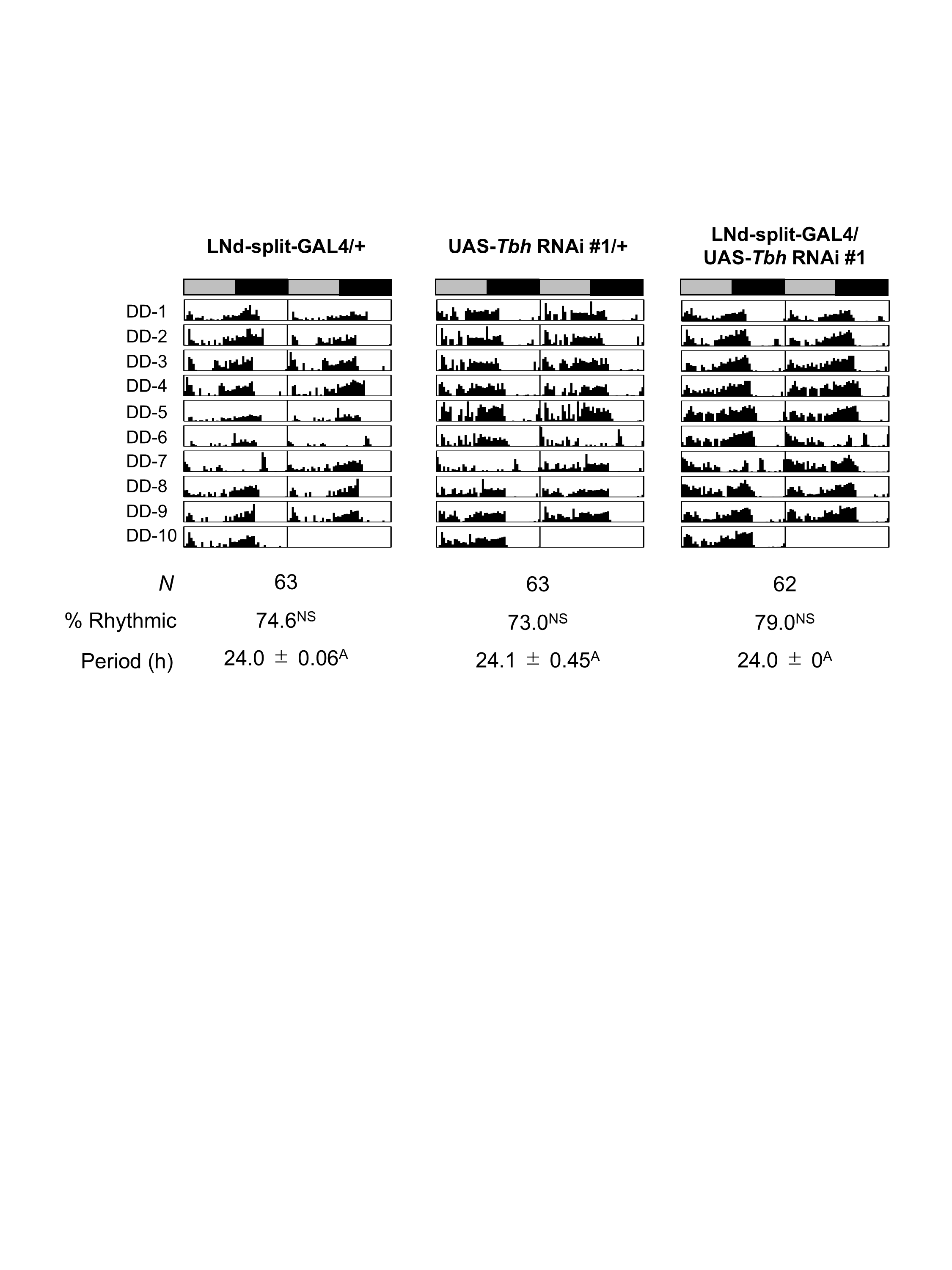

Supplement: S4 Fig — Double-plotted actogram in DD. The bar above each actogram indicates subjective day (gray) and night (black). Each actogram shows locomotor activity for 10 days in DD at 25◦C. LNd-split-GAL4/UAS-Tbh RNAi #1 males were used. LNd-split-GAL4/+ and UAS-Tbh RNAi #1/ + males were used as the control. N, sample size. For the percentage of rhythmicity (% rhythmic), UAS-Tbh RNAi #1/+ or LNd-split-GAL4/UAS-Tbh RNAi #1 males were compared with GAL4 control males. We used the Kruskal–Wallis test for comparisons of the circadian period. The same letters in superscripts indicate no significant difference (P > 0.05). N, sample size. (TIF) [file pgen.1012045.s004.TIF]

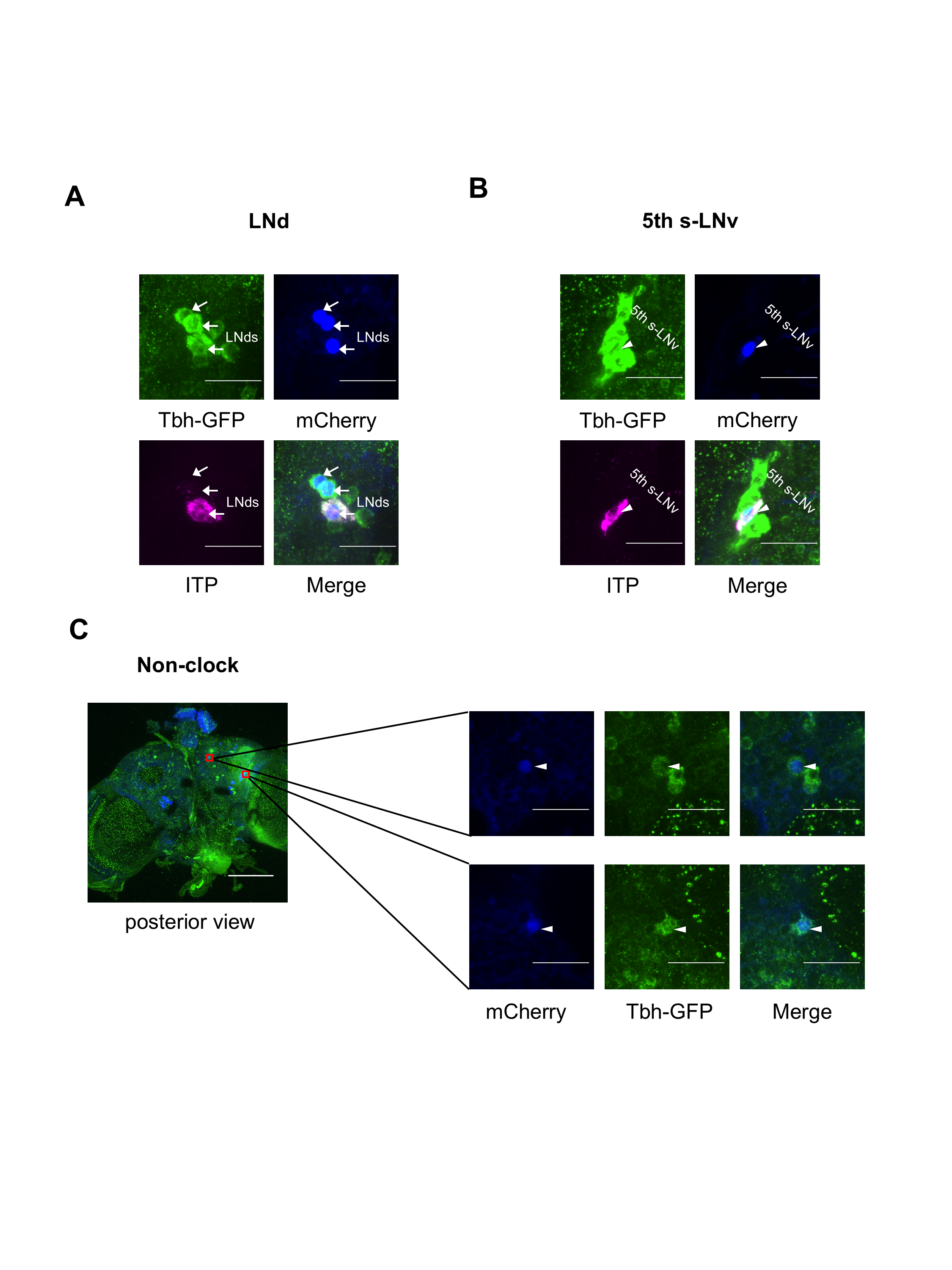

Supplement: S5 Fig — (A–C) UAS-mCherry.NLS/ + ; R78G02/Tbh-GFP males were used in the experiments. (A) Confocal section images at LNd level of the adult brain. Scale bars represent 20 μm. Arrows, LNds; Green, Tbh-GFP; Blue, mCherry.NLS; Magenta, ITP. (B) Confocal section images at 5th s-LNv level of the adult brain. Scale bars represent 20 μm. Arrow heads, 5th s-LNv; Green, Tbh-GFP; Blue, mCherry.NLS; Magenta, ITP. (C) Confocal images of posterior view of the adult brain. Scale bars represent 100 μm (left image) and 20 μm (six images on the right). Arrows, Tbh-GFP-positive non-clock neurons; blue, mCherry,NLS; green, Tbh-GFP. (TIF) [file pgen.1012045.s005.TIF]

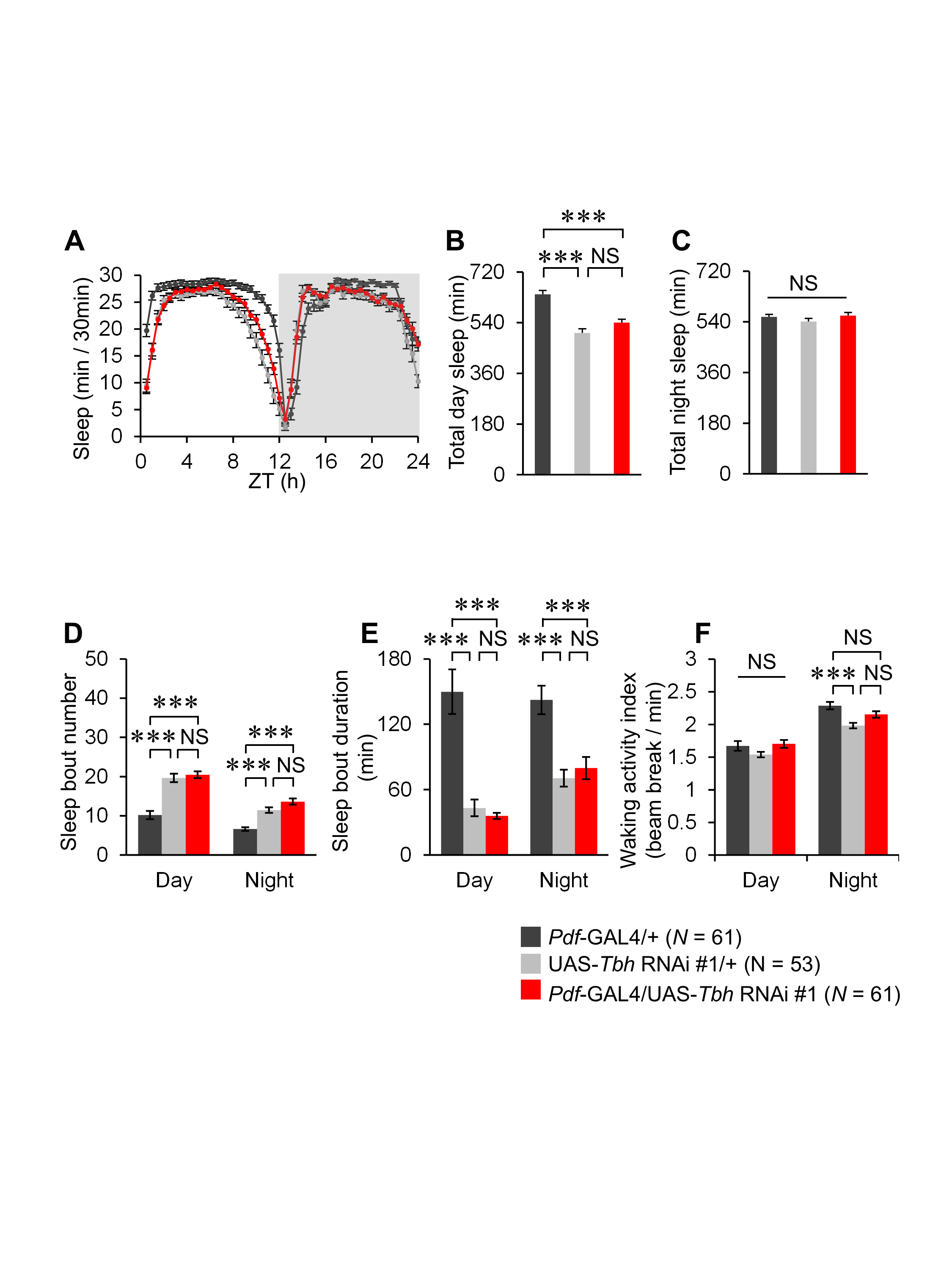

Supplement: S6 Fig — All sleep/wake parameters (daily sleep pattern, total day sleep, total night sleep, sleep bout number, sleep bout duration, waking activity index) were analyzed using the data averaged over 3 days of LD. (A–F) Error bars show SEM in each figure. Black circles and bars, Pdf-GAL4/ + ; gray circles and bars, UAS-Tbh RNAi #1/ + ; red circles and bars, Pdf-GAL4/UAS-Tbh RNAi #1. ***, P < 0.001; NS, not significant. (A) Daily sleep patterns of control and experimental flies. (B) Total sleep amount during the day. (C) Total sleep amount during the night. (D) Sleep bout number during day and night. (E) Sleep bout duration during day and night. (F) Waking activity indices during day and night. (TIF) [file pgen.1012045.s006.TIF]
