# Supplementary material for: Octopamine signaling from clock neurons plays dual roles in Drosophila long-term memory
Source: PLoS Genet. 2026 Feb 17;22(2):e1012045. doi: 10.1371/journal.pgen.1012045 (PMC12923120; doi:10.1371/journal.pgen.1012045)
Supplement: Attachment — Submitted filename: Sakei review.docx [file pgen.1012045.s008.docx]

This report continues a series of recent papers documenting molecules and cells needed for courtship suppression memory in *Drosophila*. Previously the Sakei lab identified a pair of cells in the LNd cluster (a group of neurons that are part of the central clock) as being important for courtship memory. In this report, they examine the neurotransmitters that are needed in that pair of neurons, a nearby “subcluster” (pair) of neurons, and more numerous pdf-expressing neurons in the central clock. Their data shows that there is a differential requirement for octopamine depending upon the exact cells and interval of query during the processes of memory formation, consolidation and maintenance. Specifically, octopamine is required in 2 LNd neurons for “maintenance” (late long-term memory or LTM), whereas it is required in clock neurons (defined using the pdf-Gal4 driver) for early learning/memory. The strong points of the manuscript are the cellular imaging and behavioral analyses. Using these approaches, the authors describe the necessity for octopminergic neurotransmission during two different time windows of the processing of courtship memory, and that octopamine is likely expressed in the relevant neurons. The authors validate their Gal4 drivers, show that their genetic manipulations have the expected effects on target mRNA levels, and use temporal and spatially delimited interventions to rule out effects on neurodevelopment indirectly affecting mature neuron function. Their effects are clear and their conclusions are warranted. They also carefully demonstrate that their effects are unlikely to be indirect through effects on circadian rhythmicity or sleep.

Three suggestions for the presentation and interpretation of the data.

1. In Figures 2-6, it seems like there is a labeling mistake (or I don’t understand their labeling system). The third column of each data set should be labeled + instead of -, since both transgenes are present in the third comparison. The first and second histograms are the individual parents, while the third is the progeny from the cross between the parents.

2. Although I am familiar with the Gal4 drivers and cells that the authors are using, following the data was confusing. I suggest putting the cartoon in Figure 4C in Figure 1 so that the reader meets the drivers and cells at the beginning. The current Figure 1A (summary of results) should be moved to a later Figure. Although the current order makes sense in terms of the flow of the data (especially the imaging data), it is good to see the relationship amongst the cells at the beginning.

3. For chronic disruptions (Figures 1, 4 and 5), the effect on adult behavior could be indirect through disrupting neuronal development. The authors are aware of this, since they use testing at 1h after conditioning (Supplementary Figure S5) to demonstrate that early memory is intact when pdf-Gal4 is used to drive Tbh^RNAi^. This result suggests strongly that there is no developmental effect of chronic knock-down of the synthesis of octopamine indirectly affecting adult brain function, totally validated the chronic disruption shown in Figure 5. Ideally, the authors should show a similar (non-effect on 1h memory) for Figures 1 and 4. But since there is no effect in Figure 4, there is unlikely an effect on neuronal development leading to an indirect effect on adult behavior. Therefore, this worry only applies to the data in Figure 1. If the authors have a test of 1h memory for any of the combinations used in Figure 1, adding it to the Supplementary data would be ideal. Otherwise, adding some verbiage to qualify their interpretations seems appropriate. For example, the flies show normal circadian rhythms and sleep patterns, consistent with normal neuronal development making an indirect effect less likely.

4. In the learning/memory field, the usual description of the trajectory of a memory is: learning, consolidation, maintenance, and retrieval (at the time of testing). Since a disruption prior to conditioning (Figures 2E, 3 and 4B) could affect learning, early consolidation or both, the authors should use “learning and/or consolidation” to refer to the effects of those manipulations prior to training, rather than just consolidation (Line 198). Consolidation (Line 202) should be replaced with learning or conditioning.

5. The assignment of 2d memory as a “maintenance” phase seems somewhat arbitrary. Late consolidation might be better.

To the Editor

There are two related issues that make labeling of memory phases murky.

1. The first is the phenomenon called “systems consolidation” which in mammals refer to the (apparent) “movement” of memory from one region of the brain to another. More accurately it is the apparent change in dependency upon one region (early on a requirement for the hippocampus) versus another region (later on the required region is in subregions of the cortex). Systems consolidation seems to be involved when late, long-term memories are produced which in mammalian models is usually associated with “remote memory” (>2-4 weeks after the end of training). Once more data involving other behaviors (besides fear conditioning) exists, it is likely that there will be two “types” of consolidation, synaptic and systems for most types of memories. At that point, the best descriptive terms might be: learning (de novo protein synthesis not required), synaptic consolidation, systems consolidation, maintenance and retrieval. Systems consolidation has been suggested for fly olfactory avoidance memory, although the data is very incomplete, the timing of the memory phases is greatly compressed, and the transition times are unknown. Whether systems consolidation or remote memory exists for courtship suppression is unknown. Intuitively, it seems that this behavior might **not have** remote memory since it’s existence would harm the continuation of the species.

2. Although it is intuitive that memory phases (learning, early memory, late memory) occur in series, we have quite a bit of unpublished data that suggests that memory phases can occur in parallel. The dissection of memory into different phases requires extensive knowledge about neurons that are part of different memory phases, and molecules in those neurons that contribute to distinct phases. It also requires data using loss-and/or-gain of function genetic manipulations that only affect discrete phases. For example, it is important to enhance or inhibit the early phase without affecting the later one. It is also necessary to inhibit or enhance the late one (during an early time point) and have no effect on early memory. It is possible that the parallel phases are unique to the behavior that we use (olfactory avoidance) and does not occur for courtship suppression, since its permanence would not favor species survival. Parallel memory phases may be an aspect of systems consolidation. The authors somewhat arbitrarily assign the term “maintenance” to an interval 2 days after conditioning without any real data supporting that all phases are complete.

These two issues make the labeling of memory in the manuscript somewhat naïve. Therefore I suggest more generic terms like learning/early consolidation for conditioning and later consolidation instead of maintenance.
