# Supplementary material for: Octopamine signaling from clock neurons plays dual roles in Drosophila long-term memory
Source: PLoS Genet. 2026 Feb 17;22(2):e1012045. doi: 10.1371/journal.pgen.1012045 (PMC12923120; doi:10.1371/journal.pgen.1012045)
Supplement: Attachment — Submitted filename: Responses to reviewer comments-12 22 2025.docx [file pgen.1012045.s010.docx]

**Responses to reviewers’ comments:**

First of all, we would like to thank the reviewers for their thorough and well-considered reviews. We found the reviewers' comments most helpful and have revised the manuscript accordingly. The following are our point-by-point responses to the reviewers' comments and descriptions of the changes we have made in the manuscript. The reviewers' comments are in underlined text and our responses are not underlined.

***Reviewer #1:***

**1. It is preferable to show the RNAi specificity. The authors used only 1 RNAi line in PDF neuron experiments, which can be improved if they used additional lines, like LNd. In addition, rescue experiments will also strengthen the conclusion.**

In accordance with the reviewer's comment, we have conducted additional experiments to knock down *Tbh* using UAS-*Tbh* RNAi#2. Consistent with the results obtained using UAS-*Tbh* RNAi#1, impaired LTM was also observed in *Pdf*-GAL4/UAS-*Tbh* RNAi#2 males. These results are presented in Fig. 5G of the revised manuscript.

In the original manuscript, we reported that the naive CI in *Pdf*-GAL4/UAS-*Tbh* RNAi#1 males was significantly reduced compared with that in GAL4 or UAS controls. Similarly, the naive CI in *Pdf*-GAL4/UAS-*Tbh* RNAi#2 males was also modestly but significantly reduced compared with that in controls. This result has also been added to the Results section in the revised manuscript as follows (Lines 264–269).

In Figures 5F and G, the naive CI in *Pdf*-GAL4/UAS-*Tbh* RNAi #1 and *Pdf*-GAL4/UAS-*Tbh* RNAi #2 males was significantly lower than that in GAL or UAS control (*Pdf*-GAL4/+ vs *Pdf*-GAL4/UAS-*Tbh* RNAi #1, Steel–Dwass test, *q* = 3.90, *P* < 0.001; UAS-*Tbh* RNAi #1/+ vs *Pdf*-GAL4/UAS-*Tbh* RNAi #1, Steel–Dwass test, *q* = 4.77, *P* < 0.001; *Pdf*-GAL4/+ vs *Pdf*-GAL4/UAS-*Tbh* RNAi #2, Steel–Dwass test, *q* = 2.60, *P* < 0.05; UAS-*Tbh* RNAi #2/+ vs *Pdf*-GAL4/UAS-*Tbh* RNAi #2, Steel–Dwass test, *q* = 3.14, *P* < 0.01).

In this study, we found that *Tbh* expression in both Pdf neurons and LNds is required for LTM consolidation and maintenance. Thus, inducing *Tbh* expression only in Pdf neurons of *Tbh* knockout mutants is not sufficient to maintain LTM, and the LTM impairment are not expected to be fully restored. Consequently, gene rescue experiments were not carried out in this study.

**2. As for R78G02 experiments, the authors guessed the involvement of non-clock neurons. This conclusion solely depends on the results of R54D11 and LNd-split GAL4 experiments. However, the efficiencies of RNAi by different GAL4 are different, even they are expressed in the same cells. Therefore, the results can be interpreted in another way. It is also preferable to use more specific GAL4 drivers, which show positive effects in memory functions.**

We searched for publications describing the *R78G02*-positive and Tbh-positive non-clock neurons identified in this study; however, we were unable to find any such reports. Therefore, it is most likely that the functions of these neurons have not yet been characterized. Consequently, we were unable to use specific GAL4 lines targeting these neurons in this study. If such GAL4 lines become available in the future, we plan to perform follow-up analyses to elucidate the properties of these neurons and their roles in regulating LTM.

As the reviewer noted, the efficiency of RNAi depends on the level of GAL4 expression. Among the GAL4 lines used in this study (*R78G02*, *R54D11*, and LNd-split-GAL), we have the impression that LNd-split-GAL4 appears to have the weakest GAL4 expression. Nevertheless, LTM impairments were confirmed upon Tbh knockdown. Therefore, we believe it is likely that *R54D11*-positive cells are not involved in LTM, and *R78G02*-positive and Tbh-positive non-clock neurons are likely involved in LTM consolidation.

**3. The authors described that the baseline of the courtship index (naïve CI) were different between LNd-split-GAL4/UAS-Tbh RNAi “1 and Pdf-GAL4/UAS-Tbh RNAi #1. Since memory index of LTM is affected by the native CI, they should mention how this difference may affect the results in the discussion.**

In the original manuscript, we showed that *Tbh* knockdown in LNds and Pdf-neurons reduces, to a greater or lesser extent, courtship activity in naive males. The mean naive CI in LNd-split-GAL4/UAS-*Tbh* RNAI #1 males was approximately 62%, and that in *Pdf*-GAL4/UAS-*Tbh* RNAI #1 males was approximately 50%. However, we reviously confirmed that courtship suppression via courtship conditioning occurs even in the wild-type strain with a very low naive CI (OGS-4 strain, mean CI = 26.1%; #1619 strain, mean CI = 37.7%) (Sakai et al., 2004, *PNAS* 101: 16058-16063.). Therefore, it is unlikely that a low baseline level of courtship activity alone would prevent the detection of courtship memory.

In the revised manuscript, the following text has been added to the Results section (Lines 142–148).

As shown in Figure 1C, the naive CI in LNd-split-GAL4/UAS-*Tbh* RNAi #1 males (mean naive CI = 62%) was significantly lower than that in LNd-split-GAL4 control males (Steel–Dwass test, *q* = 3.338, *P* < 0.020), despite the absence of a significant difference in the naïve CI between LNd-split-GAL4/+ and UAS-*Tbh* RNAi #1/+ males (Steel–Dwass test, *q* = 1.60, *P* = 0.795). We previously confirmed that the wild-type strains with a mean naive CI of less than 40% exhibit courtship suppression on day 5 after 7 h conditioning [6]. Thus, it is unlikely that the difficulty in detecting courtship memory in LNd-split-GAL4/UAS-*Tbh* RNAi #1 males is solely due to a reduction in naive CI.

A reduction in naive CI caused by Pdf-neuron-specific *Tbh* knockdown was observed only in F_1_ males between *Pdf*-GAL4 and UAS-*Tbh* RNAi. In the temporal knockdown experiments, naive CI was not reduced, although an LTM defect was detected. Therefore, we believe that whether Tbh affects LTM should be discussed on the basis of the results shown in Figure 6.

Regarding Figures 5E–G, we think it is preferable to describe the results objectively. The description of the results of Pdf-neuron-specific *Tdc2* or *Tbh* knockdown in Figs. 5E and F in the original manuscript was as follows: The knockdown of *Tdc2* or *Tbh* in Pdf neurons impaired LTM (Figs 5E and F). However, in the revised manuscript, we have described the results as follows (Lines 262–264).

In males with knockdown of *Tdc2* or *Tbh* in Pdf neurons, MI was significantly reduced compared with those in GAL4 and UAS control males (Figs 5E–G).

**4. The authors concluded Tbh knockdown did not affect the circadian rhythm and sleep. But in the supplemental figures (S2, S6), there are minor changes in a couple of parameters. Thus, they should rephrase, maybe “There were minor effects which are not likely to affect LTM.”**

Although the reviewer pointed out minor changes in a couple of parameters in Figs. S2 and S6, these may have resulted from his/her misinterpretation.

In Fig. S2, only daytime waking activity (Fig. S2F in the original manuscript) was elevated by LNd-specific *Tbh* knockdown. For the other sleep parameters, either no significant differences were detected between the UAS control and *Tbh* knockdown flies, or the values of *Tbh* knockdown flies fell between those of the GAL4 and UAS controls. Thus, we did not obtain any results indicating changes in sleep quantity or quality due to *Tbh* knockdown. The increase in waking activity is described in the Results section of the original manuscript. Moreover, no clear abnormalities in other sleep parameters were observed following *Tbh* knockdown. Therefore, in the Result section in the original manuscript, we stated as follows: *Tbh* knockdown has little effect on the amount and quality of sleep.

For all sleep parameters shown in Fig. S6, no significant differences were found between *Tbh* knockdown flies and UAS controls. Again, we obtained no evidence that *Tbh* knockdown altered sleep quantity or quality. Therefore, in the Result section in the original manuscript, we stated as follows: Octopamine signaling in Pdf neurons does not appear to have a major impact on sleep phenotypes or spontaneous activity.

In the discussion part of the revised manuscript, we have made the following corrections (Lines 334-336).

Thus, we demonstrated for the first time that octopamine synthesis in clock neurons is essential for courtship LTM without significantly affecting circadian behavioral rhythms and sleep in *Drosophila*.

***Reviewer #2:***

**1. In Figures 2-6, it seems like there is a labeling mistake (or I don’t understand their labeling system). The third column of each data set should be labeled + instead of -, since both transgenes are present in the third comparison. The first and second histograms are the individual parents, while the third is the progeny from the cross between the parents.**

Thank you for pointing out the labeling errors. We have corrected them all in the revised manuscript.

**2. Although I am familiar with the Gal4 drivers and cells that the authors are using, following the data was confusing. I suggest putting the cartoon in Figure 4C in Figure 1 so that the reader meets the drivers and cells at the beginning. The current Figure 1A (summary of results) should be moved to a later Figure. Although the current order makes sense in terms of the flow of the data (especially the imaging data), it is good to see the relationship amongst the cells at the beginning.**

We have revised the manuscript iin accordance with the reviewer's comments.

**3. For chronic disruptions (Figures 1, 4 and 5), the effect on adult behavior could be indirect through disrupting neuronal development. The authors are aware of this, since they use testing at 1h after conditioning (Supplementary Figure S5) to demonstrate that early memory is intact when pdf-Gal4 is used to drive Tbh^RNAi^. This result suggests strongly that there is no developmental effect of chronic knock-down of the synthesis of octopamine indirectly affecting adult brain function, totally validated the chronic disruption shown in Figure 5. Ideally, the authors should show a similar (non-effect on 1h memory) for Figures 1 and 4. But since there is no effect in Figure 4, there is unlikely an effect on neuronal development leading to an indirect effect on adult behavior. Therefore, this worry only applies to the data in Figure 1. If the authors have a test of 1h memory for any of the combinations used in Figure 1, adding it to the Supplementary data would be ideal. Otherwise, adding some verbiage to qualify their interpretations seems appropriate. For example, the flies show normal circadian rhythms and sleep patterns, consistent with normal neuronal development making an indirect effect less likely.**

In accordance with reviewer's comment, we tested whether the LNd-specific knockdown of *Tbh* affects STM (1 h memory) using LNd-split-GAL4/UAS-*Tbh* RNAi#1 males. We confirmed that *Tbh* knockdown in LNds did not impair STM. This finding has been included in the revised manuscript (Lines 139–140, S2A Fig).

**4. In the learning/memory field, the usual description of the trajectory of a memory is: learning, consolidation, maintenance, and retrieval (at the time of testing). Since a disruption prior to conditioning (Figures 2E, 3 and 4B) could affect learning, early consolidation or both, the authors should use “learning and/or consolidation” to refer to the effects of those manipulations prior to training, rather than just consolidation (Line 198). Consolidation (Line 202) should be replaced with learning or conditioning.**

We have revised the manuscript iin accordance with the reviewer's comments.

**5. The assignment of 2d memory as a “maintenance” phase seems somewhat arbitrary. Late consolidation might be better.**

Researches in recent studies on olfactory aversive memory in *Drosophila* have proposed a model suggesting that two phases exist in LTM [LTM and late phase LTM (LP-LTM) in Figure A] (Davis 2011, 2023). LTM formation depends on gene expression mediated by the transcription factor CREB and on *de novo* protein synthesis (Tully et al., 1994; Yin et al., 1994). Recent studies focusing on memory trace reveal that traces of LTM are formed in MB α neurons within at least 24 hours after conditioning, and that subsequent LP-LTM traces are maintained in MB γ neurons from 18 to 48 hours after conditioning (see Figure A).

Research on courtship memory also indicates that LTM is CREB-dependent (Sakai et al., 2004; Inami et al., 2021). However, reports on the cellular traces of courtship memory using *in vivo* imaging remain extremely limited. Consequently, it is still unclear whether a memory phase equivalent to the LP-LTM observed in olfactory memory also exists in courtship memory. Genetic studies of courtship memory have shown that the molecular and cellular mechanisms underlying memory one day after conditioning (1 d memory) differ from those two days after conditioning (2 d memory) (see Inami et al., 2022). For example, CREB activity in MB αβ and γ neurons during conditioning is essential to establish courtship LTM, whereas after the second day of conditioning, CREB activity in only MB αβ neurons is necessary for the persistence of LTM. (Inami et al., 2021). On the basis of these findings, we have referred to the period up to the day after conditioning as the “consolidation phase,” and the period from the second day onward as the “maintenance phase” (see Figure B). Given the background of this research on courtship memory, in this paper, we also use similar terminologies. As research on the cellular traces of courtship memory advances, it may become necessary to redefine whether the 2 d memory represents LP-LTM or the LTM maintenance phase. At present, however, we believe it is still premature to make such a distinction.

The original manuscript provided insufficient explanation of the terms “consolidation” and “maintenance” in courtship memory research. Therefore, we have added the following explanation to the introduction section (Lines 60–64) of the revised manuscript.

Genetic studies of courtship LTM have pointed out that the molecular and cellular mechanisms underlying memory one day after conditioning differ from those two days after conditioning [3, 7]. Therefore, the period up to the day after conditioning has been conceptually termed the LTM consolidation phase, whereas the period from the second day onward has been termed the LTM maintenance phase [3, 7]. The same terminologies are used in this paper.
